# Psychological capital and music-related burnout among university music students: the mediating effects of meaning in music and flow experience

**DOI:** 10.3389/fpsyg.2026.1956838

**Published:** 2026-10-14

**Authors:** Xin Zhang, Chuanwen Yu

**Affiliations:** 1 School of Music and Dance, Heze University, Heze, Shandong, China; 2 School of Physical Education and Health, Heze University, Heze, Shandong, China

**Keywords:** flow experience, meaning in music, music-related burnout, psychological capital, university music students

## Abstract

**Introduction:**

University music students face intensive practice, repeated assessment, public performance, competition, and career uncertainty, which may increase their vulnerability to music-related burnout. Drawing on positive psychology and resource-based perspectives, this study examined the association between music-specific psychological capital and music-related burnout and investigated the mediating roles of meaning in music and flow experience.

**Methods:**

A cross-sectional survey was conducted with 565 music students from 10 universities in Shandong Province, China. Structural equation modelling and bootstrapping analyses were used to examine the hypothesized relationships among psychological capital, meaning in music, flow experience, and music-related burnout.

**Results:**

Psychological capital was positively associated with meaning in music and flow experience, while both meaning in music and flow experience were negatively associated with music-related burnout. Although the direct association between psychological capital and burnout was not significant, significant indirect associations were identified through meaning in music, flow experience, and the sequential pathway from meaning in music to flow experience.

**Discussion:**

These findings suggest a potential purpose-to-absorption pathway linking psychological capital with lower music-related burnout. The study extends predominantly risk-oriented research on burnout in higher music education by highlighting meaning in music and flow experience as important positive psychological processes. Interventions that strengthen psychological resources, clarify musical purpose, and foster conditions conducive to flow may be beneficial. Longitudinal and intervention studies are needed to establish temporal and causal relationships.

## Introduction

University music study typically requires extensive individual practice, ensemble rehearsal, academic coursework, auditions, examinations, competitions, and public performance. In addition to developing technical and artistic skills, music students must navigate ongoing teacher evaluation, peer comparison, high performance expectations, physical demands, and career uncertainty (Jääskeläinen, 2022). These recurring demands can accumulate over time, particularly when students have limited psychological resources or opportunities for recovery. Existing literature indicates that music students' workload and wellbeing are shaped by interacting personal, pedagogical, institutional, and career-related pressures (Jääskeläinen et al., 2023; Rosset et al., 2022). Prolonged exposure to such demands may contribute to music-related burnout. In the present study, music-related burnout refers to a domain-specific pattern of emotional and physical exhaustion, psychological distancing from music-related activities, and diminished musical efficacy. Unlike transient fatigue following intensive practice or performance, burnout reflects a more sustained decline in energy, motivation, perceived competence, and emotional connection to music. It may undermine students' practice quality, artistic development, academic persistence, and long-term commitment to a music career. Although music-related burnout among university music students has received limited direct attention, available evidence indicates that burnout, disengagement, and diminished accomplishment are relevant concerns in high-level music training (Zabuska et al., 2018).

Research on music students' mental health has primarily emphasized risk factors, including excessive workload, performance anxiety, perfectionism, physical pain, competitive pressure, and maladaptive coping (Barros et al., 2022). Comparatively less attention has been paid to positive, potentially malleable psychological resources that may help sustain musical engagement. One such resource is psychological capital, a higher-order positive psychological state comprising self-efficacy, hope, resilience, and optimism (Luthans et al., 2007b). These resources may help students remain confident in demanding musical situations, sustain motivation toward valued goals, identify alternative pathways when obstacles arise, recover from criticism and disappointing performances, and maintain positive expectations for future development. Evidence from broader higher-education settings suggests that psychological capital is associated with greater academic engagement and lower academic burnout (Barratt and Duran, 2021; Wang et al., 2021). However, psychological capital is a relatively general personal resource, whereas music-related burnout emerges within students' experiences and appraisals of music learning, practice, rehearsal, and performance. Its relevance to burnout may therefore depend on whether these broader psychological resources are translated into adaptive, domain-specific forms of musical engagement.

This study focuses on two such domain-specific processes: meaning in music and flow experience. Meaning in music refers to the extent to which students experience their musical engagement as purposeful, personally significant, identity-relevant, and aligned with valued life or career goals. For many university students, music is not merely an academic subject but also an important source of self-expression, identity, belonging, and anticipated future development. Evidence indicates that music students' workload experiences are closely related to the meanings they attribute to their musical participation (Jääskeläinen, 2023). Perceiving practice and performance as purposeful may help students sustain motivation and emotional commitment despite repetition, frustration, and slow progress. By contrast, activities perceived as externally imposed or disconnected from personal values may become increasingly draining.

Flow experience represents a more immediate form of optimal engagement characterized by focused attention, deep absorption, perceived control, fluent action, and intrinsic enjoyment. Music practice and performance may be particularly conducive to flow because they involve clear goals, continuous feedback, embodied skill, and ongoing adjustment between task demands and skill level. Studies of musicians and music students have identified flow as an important aspect of positive musical engagement, although its occurrence may vary with performance anxiety, audience presence, perceived challenge, and perceived competence (Antonini Philippe et al., 2022; Guyon et al., 2022; Spahn et al., 2021). A systematic review likewise found an overall negative association between flow and burnout symptoms while emphasizing that causal inference remains limited by the predominance of cross-sectional evidence (Aust et al., 2022). Meaning in music and flow experience are related but conceptually distinct: meaning concerns why musical engagement is worth pursuing, whereas flow concerns how that engagement is experienced in the moment. A stronger sense of meaning may support sustained attention and effort, thereby increasing opportunities for flow; flow, in turn, may make practice, rehearsal, and performance more intrinsically rewarding and reinforce perceived musical competence.

Recent research found that self-esteem and flow sequentially mediated the relationship between psychological capital and music performance anxiety among Chinese university music students (Jiang and Tong, 2024). However, music performance anxiety primarily reflects distress in evaluative performance situations, whereas music-related burnout represents a broader and more enduring pattern of exhaustion, distancing, and diminished efficacy. In addition, self-esteem is a relatively general evaluation of self-worth, whereas meaning in music reflects the domain-specific purpose, personal significance, and identity relevance of musical engagement. It therefore remains unclear whether psychological capital is associated with music-related burnout through a theoretically distinct pathway involving meaning in music and flow experience. Accordingly, the present study examined the association between psychological capital and music-related burnout among university music students and tested the specific and sequential indirect roles of meaning in music and flow experience. The study extends the literature by shifting attention from predominantly risk-oriented accounts of music-related burnout toward domain-specific positive psychological processes and by evaluating a purpose-to-absorption pathway in which psychological capital is associated with stronger meaning in music, greater flow experience, and lower music-related burnout. The findings may provide a theoretical basis for interventions that combine psychological-capital development with strategies to strengthen musical purpose and create conditions conducive to optimal engagement.

## Literature review and hypothesis development

### Psychological capital and meaning in music

Psychological capital is a state-like, higher-order positive psychological resource comprising self-efficacy, hope, resilience, and optimism (Eid et al., 2026). These resources influence how individuals appraise demanding circumstances, pursue goals, respond to setbacks, and maintain positive expectations. Psychological capital may be particularly relevant for university music students, whose development depends on prolonged practice, repeated performance, and sustained investment despite uncertain outcomes. Music students regularly encounter slow progress, critical feedback, performance errors, competition, and career uncertainty. How they appraise these experiences may shape whether musical engagement continues to feel worthwhile. Meaning in music refers to the extent to which students experience music learning, practice, rehearsal, and performance as purposeful, personally significant, identity-relevant, and connected to valued future goals. Music may serve as an important source of self-expression, identity, belonging, and career aspiration. Evidence suggests that music students' workload experiences are closely linked to the meaning they attribute to musical activity (Jääskeläinen, 2023). Psychological capital may support meaning in music by helping students interpret obstacles as opportunities for development, connect daily practice with long-term goals, maintain a sense of purpose after setbacks, and sustain positive expectations about musical growth. Students with higher psychological capital may therefore be more likely to view music-related demands as meaningful investments rather than externally imposed burdens.

H1. Psychological capital is positively associated with meaning in music among university music students.

### Psychological capital and flow experience

Flow refers to an optimal experiential state characterized by intense concentration, deep absorption, fluent action, perceived control, and intrinsic enjoyment (Waterman, 2025). Music practice and performance may be conducive to flow because they involve clear goals, immediate feedback, embodied skill, and continuous calibration between challenge and skill (Zielke et al., 2023). However, flow depends in part on how students appraise and respond to task demands. Challenges appraised as threatening may increase anxiety, self-monitoring, and attentional disruption, whereas challenges perceived as manageable may foster concentration and absorption. Psychological capital may facilitate flow by strengthening perceived control, sustaining goal-directed attention, supporting recovery after errors, and maintaining positive expectations.

H2. Psychological capital is positively associated with flow experience among university music students.

### Psychological capital and music-related burnout

Music-related burnout is a domain-specific syndrome characterized by persistent exhaustion, psychological distancing from music-related activities, and diminished musical efficacy. It differs from transient fatigue in that it reflects a more sustained loss of energy, enthusiasm, emotional connection, and perceived competence. Recent evidence suggests that fatigue, disengagement, and reduced achievement are relevant concerns among students engaged in intensive music training (Zhu, 2025). Conservation of resources theory proposes that burnout becomes more likely when valued resources are lost, threatened, or insufficiently replenished (Hobfoll et al., 2018). Psychological capital may function as a personal resource that helps students regulate negative emotions, persist through difficulties, recover from setbacks, and interpret demanding experiences more constructively. Evidence from broader higher-education contexts links higher psychological capital with lower academic burnout and greater engagement (Gebregergis and Csukonyi, 2024). Psychological capital does not remove the objective demands of music education, but it may influence whether those demands are appraised as overwhelming or manageable. Students with higher psychological capital may therefore report less exhaustion, psychological distancing, and diminished musical efficacy.

H3. Psychological capital is negatively associated with music-related burnout among university music students.

### The mediating roles of meaning in music and flow experience

Meaning in music may represent one pathway through which psychological capital is associated with burnout. Psychological capital may help students connect routine practice, feedback, and setbacks with valued artistic and professional goals. Musical activities may become increasingly depleting when they are perceived as meaningless, coercive, or disconnected from personal values. By contrast, perceiving music as meaningful may sustain motivation and emotional investment while placing short-term difficulties within a broader developmental context. Evidence from workplace research likewise indicates that meaningful engagement is negatively associated with burnout (Allan et al., 2019).

H4a. Psychological capital is indirectly associated with music-related burnout through meaning in music, such that higher psychological capital is associated with stronger meaning in music, which in turn is associated with lower music-related burnout.

Flow may represent a second pathway through which psychological capital is associated with burnout. Psychological capital may support sustained attention, perceived control, and challenge–skill balance, all of which are central to flow. In turn, flow may make practice and performance more enjoyable and fulfilling and may therefore be associated with lower burnout. This proposed association is conceptually plausible because burnout is characterized by exhaustion, psychological distancing, and diminished efficacy, whereas flow involves absorption, enjoyment, and perceived competence (Habe et al., 2019). Consistent with this account, a systematic review found that flow was generally negatively associated with burnout symptoms, although the direction of causality remains uncertain (Aust et al., 2022).

H4b. Psychological capital is indirectly associated with music-related burnout through flow experience, such that higher psychological capital is associated with greater flow experience, which in turn is associated with lower music-related burnout.

### The sequential mediating roles of meaning in music and flow experience

Meaning in music and flow experience represent distinct but closely related aspects of musical engagement. Meaning concerns why students consider music worth pursuing, whereas flow concerns how engagement is experienced in the moment. Meaning can therefore be understood as a broader motivational appraisal, while flow represents a more immediate experiential state. Students who experience music as personally meaningful may be more willing to sustain attention, effort, and emotional investment in practice and performance. Such investment may support conditions conducive to flow, including sustained concentration, perceived control, and a balance between challenge and skill. Conversely, musical activities perceived as externally imposed or disconnected from personal goals may evoke resistance, distraction, and self-monitoring, thereby reducing opportunities for absorption. Psychological capital may initiate this sequence by supporting more constructive and purposeful appraisals of musical engagement. Meaning in music may then provide a motivational foundation for flow, which may in turn be associated with lower burnout through greater intrinsic satisfaction, perceived competence, and emotional connection to music. This proposed pathway extends previous research showing that self-esteem and flow sequentially linked psychological capital with music performance anxiety (Jiang and Tong, 2024) by examining a distinct purpose-to-absorption process in relation to music-related burnout. The hypothesized relationships are presented in Figure 1.

**Figure 1:**
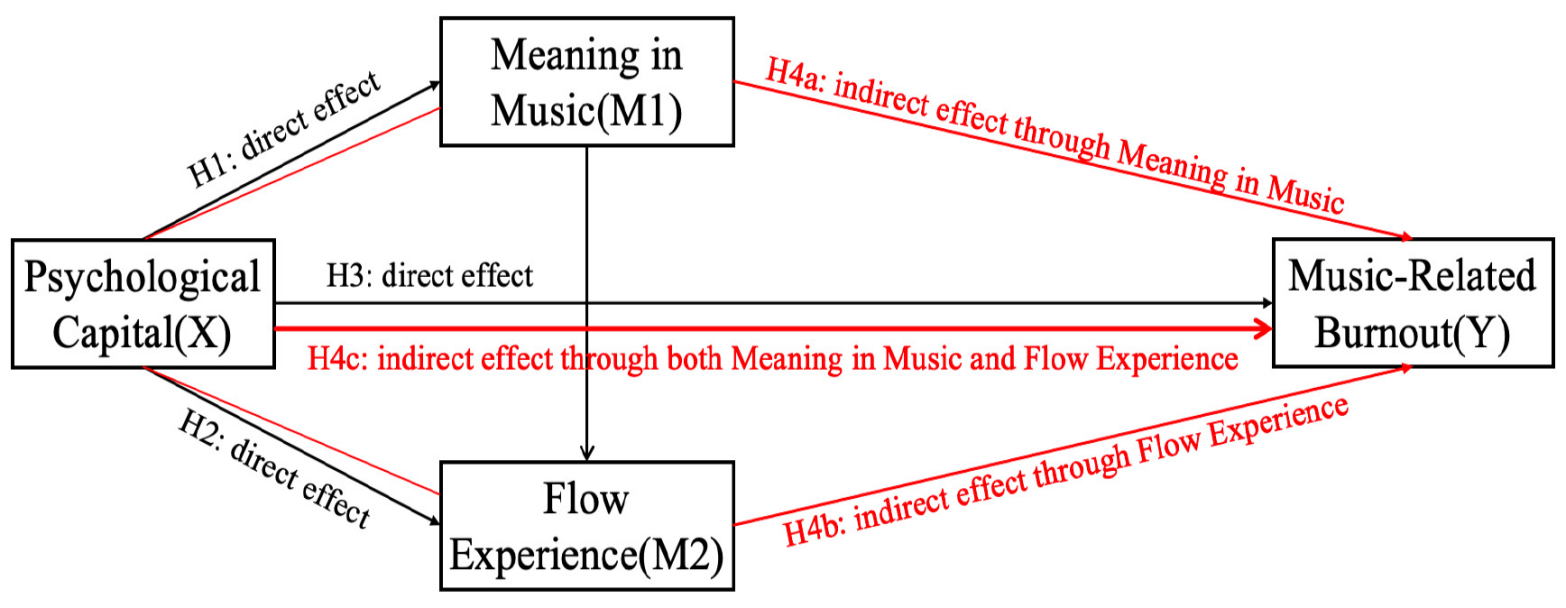
Conceptual model and research hypotheses.

H4c. Psychological capital is indirectly associated with music-related burnout through the sequential pathway of meaning in music and flow experience.

## Methods

### Study design and participants

A cross-sectional questionnaire study was conducted from March 1 to June 30, 2026, among students enrolled in university-level music programs at 10 universities in Shandong Province, China. Participants were recruited through convenience sampling with support from the participating universities. Eligible participants were undergraduate or postgraduate students in music-related programs who regularly engaged in music learning, individual practice, rehearsal, assessment, or performance. As shown in Table 1, 600 questionnaires were distributed. After data screening, 35 responses were excluded because they were incomplete, contained responses outside the permissible range, were duplicate submissions, or met the prespecified criteria for careless responding. The final analytic sample comprised 565 valid responses, corresponding to a valid response rate of 94.17%. Of these participants, 176 were male (31.2%) and 389 were female (68.8%).

**Table 1:** Demographic and Music-Training Characteristics of the Participants (*N* = 565).

Characteristic	Category	*n*	%
**Gender**	Male	176	31.2
	Female	389	68.8
**Educational level**	Undergraduate Year 1	163	28.8
	Undergraduate Year 2	232	41.1
	Undergraduate Year 3	114	20.2
	Undergraduate Year 4	44	7.8
	Master's student or above	12	2.1
**Years of formal music training**	Less than 1 year	78	13.8
	1–2 years	153	27.1
	2–3 years	291	51.5
	More than 3 years	43	7.6
**Average daily practice time (past 4 weeks)**	Less than 1 h	78	13.8
	1–2 h	148	26.2
	2–3 h	149	26.4
	3–4 h	128	22.7
	More than 4 h	62	11.0
**Public performances, competitions, or professional examinations (past 12 months)**	0	29	5.1
	1–3	125	22.1
	4–6	391	69.2
	More than 7	20	3.5

### Procedure

Ethical approval was obtained from the Research Ethics Review Committee of Heze University (approval no. HZXY-2026-717) before data collection. The study was conducted in accordance with the Declaration of Helsinki. Before accessing the questionnaire, all participants received an electronic information sheet describing the study purpose, procedures, confidentiality provisions, voluntary nature of participation, and participant rights. Electronic informed consent was obtained before participants began the survey, and participants could withdraw at any time before submitting their responses without penalty. The online survey took approximately 15 min to complete. Participants were instructed to respond based on their usual experiences of music learning, individual practice, rehearsal, examinations, competitions, and public performance. The questionnaire first collected demographic and music-training information, followed by measures of psychological capital, meaning in music, flow experience, and music-related burnout. Parallel Chinese and English versions were provided. Data were collected anonymously, and no personally identifiable information was retained in the analytic dataset.

### Measures

All substantive items were rated on a 5-point scale from 1 (“strongly disagree”) to 5 (“strongly agree”). Higher scores indicated higher levels of the corresponding construct. Scale scores for the descriptive and correlational analyses were calculated by averaging the relevant items. Item-level indicators were used in the confirmatory factor analyses and structural equation models.

### Instrument translation, contextual adaptation, and preliminary validation

Instrument translation and contextual adaptation followed a multiphase process. First, two bilingual researchers with backgrounds in psychology and music education independently translated all source items into Chinese. Differences between the translations were resolved by consensus, with attention to semantic, conceptual, and contextual equivalence. A third bilingual translator, who was blinded to the original instruments, then back-translated the Chinese versions into English. The research team reviewed and resolved discrepancies between the back-translated and original items. Second, five experts with expertise in positive psychology, psychometrics, music education, and musicians' health evaluated the items for relevance, clarity, cultural appropriateness, and construct representativeness. Based on their feedback, ambiguous or contextually inappropriate wording was revised without altering the conceptual meaning or scoring direction. Third, members of the target population reviewed the items to identify difficulties in comprehension, interpretation, and response selection; their feedback informed the final questionnaire. Before the main study, the questionnaire was pilot-tested for item presentation, consistency of the response format, scoring accuracy, and administrative feasibility. The item structure, response options, construct framework, and scoring principles were retained, with changes limited to linguistic clarification and contextualization for music students.

### Psychological capital measure

Psychological capital was assessed using a 16-item music-contextualized adaptation of the Chinese Positive Psychological Capital Questionnaire, informed by the Psychological Capital Questionnaire developed by Luthans et al. (2007a) and prior application in Chinese samples (Liang et al., 2018). The adapted measure retained four theoretically specified dimensions, each represented by four items: musical self-efficacy, musical hope, musical resilience, and musical optimism. Generic work- or study-related references were replaced with references to music learning, individual practice, rehearsal, assessment, and performance while preserving the psychological content of the source items. Musical self-efficacy reflected confidence in managing technical, expressive, academic, and performance-related demands; musical hope reflected goal-directed motivation and perceived pathways toward musical goals; musical resilience reflected the capacity to recover from mistakes, criticism, and unsuccessful performances; and musical optimism reflected positive expectations regarding future musical development. An overall score was calculated by averaging the 16 items, with higher scores indicating greater music-specific psychological capital. In the measurement model, the four dimensions were specified as first-order latent factors loading onto a second-order psychological capital factor. Because the items were contextualized to the music domain, reliability and factorial validity were evaluated empirically in the present sample rather than assumed from the source instrument. The scale demonstrated excellent internal consistency and satisfactory convergent validity (Cronbach's α = 0.913, McDonald's ω = 0.918, CR = 0.921, AVE = 0.594).

### Meaning in music measure

Meaning in music was assessed using a brief four-item domain-specific measure developed for the present study rather than by directly reproducing a single existing scale. Item content was informed by research on meaningful engagement and musical identity, including the positive-meaning and meaning-making components of the Work and Meaning Inventory (Steger et al., 2012) and research on musical identity (Burland et al., 2022). The four items represented purpose (“Music gives my study and daily practice a clear sense of purpose”), personal significance (“I feel that my engagement in music is personally meaningful”), identity and values (“Music helps me understand who I am and what I value”), and alignment with valued life goals (“My music study is connected to important goals in my life”). The four items were averaged to obtain an overall score, with higher scores indicating greater perceived meaning in music. Given its brevity and domain-specific construction, the measure should be regarded as a brief index of perceived meaning in music rather than as a substitute for a comprehensive measure of meaningfulness or musical identity. Its proposed unidimensional structure was evaluated empirically in the present sample. The measure demonstrated high internal consistency and satisfactory convergent validity (Cronbach's α = 0.905, McDonald's ω = 0.907, CR = 0.907, AVE = 0.710).

### Flow experience measure

Flow experience was assessed using a brief four-item music-specific measure designed for university music students and informed by selected components of the Short Dispositional Flow Scale-2 (Jackson et al., 2008) and previous applications of flow measures among musicians and music students (Sinnamon et al., 2012; Wrigley and Emmerson, 2013). The items represented four core experiential features relevant to music practice, rehearsal, and performance: absorption (“When I practice, rehearse, or perform music, I can become fully absorbed in what I am doing”), focused attention (“During music activities, my attention is strongly focused on the music itself”), fluent action (“When I am engaged in music, my actions and musical expression seem to flow smoothly”), and perceived challenge–skill balance (“I often feel that my musical skills and the challenges I face are well matched”). The four items were averaged to obtain an overall score, with higher scores indicating greater flow experience. This brief measure does not assess all nine dimensions represented in comprehensive flow instruments such as the DFS-2 and therefore should not be interpreted as a multidimensional assessment of flow. Rather, it captures core experiential features of music-related flow relevant to students' common musical activities. Its proposed unidimensional structure was evaluated empirically in the present sample and demonstrated good internal consistency and convergent validity (Cronbach's α = 0.888, McDonald's ω = 0.889, CR = 0.889, AVE = 0.667).

### Music-related burnout measure

Music-related burnout was assessed using a 15-item music-contextualized adaptation of the Athlete Burnout Questionnaire (ABQ; Raedeke and Smith, 2001). The ABQ was selected because its three core dimensions have conceptually relevant counterparts in intensive music training. Emotional and physical exhaustion corresponds to fatigue and depletion associated with prolonged practice, rehearsal, assessment, and performance; devaluation corresponds to psychological distancing from or disengagement with musical activities; and reduced accomplishment corresponds to a diminished sense of musical competence or efficacy. Although music and athletic training are distinct, both involve prolonged skill development, repetitive practice, frequent evaluation, performance pressure, and sustained physical and psychological investment. Instrumental performance may also require substantial sensorimotor and muscular coordination, particularly during repetitive and technically demanding practice and performance. Accordingly, sport-specific references in the original items were replaced with corresponding references to music learning, practice, rehearsal, assessment, and performance while preserving the conceptual meaning and scoring direction of the original dimensions.

The adapted scale retained three dimensions, each comprising five items: music-related exhaustion, music-related cynicism, and diminished musical efficacy. Music-related exhaustion reflects fatigue and depletion arising from sustained musical demands; music-related cynicism reflects loss of interest, psychological distancing, and disengagement from musical activities; and diminished musical efficacy reflects a reduced sense of competence and accomplishment in music. Higher scores indicate greater music-related burnout. The overall scale demonstrated excellent internal consistency (Cronbach's α = 0.925, McDonald's ω = 0.925, CR = 0.925). The three dimensions also demonstrated satisfactory reliability and convergent validity: music-related exhaustion (α = 0.888, ω = 0.888, CR = 0.888, AVE = 0.612), music-related cynicism (α = 0.900, ω = 0.901, CR = 0.901, AVE = 0.645), and diminished musical efficacy (α = 0.884, ω = 0.885, CR = 0.885, AVE = 0.606). Supplementary Table 1 reports McDonald's ω, composite reliability, and average variance extracted for each dimension.

### Data analysis

IBM SPSS Statistics 26.0 and AMOS 28.0 were used for data analysis. All statistical tests were two-tailed, with statistical significance set at *p* < 0.05. The final analytic dataset comprised 565 complete cases. Descriptive statistics and Pearson correlations were calculated first. Before testing the structural model, the psychometric properties of the adapted instruments were evaluated. The sample was randomly divided, with stratification by gender and educational stage, into an exploratory subsample (n = 283) and a confirmatory subsample (*n* = 282). In the exploratory subsample, exploratory factor analysis (EFA) was conducted using principal-axis factoring with oblique (Promax) rotation. In the confirmatory subsample, confirmatory factor analysis (CFA) was used to test the retained factor structures. The measurement model included second-order psychological capital and music-related burnout factors together with the empirically supported structures for meaning in music and flow experience. Model fit was evaluated using χ^2^, CFI, TLI, RMSEA with its 90% confidence interval, and SRMR. Internal consistency was assessed using Cronbach's α and McDonald's ω. Convergent validity was examined using standardized factor loadings, composite reliability (CR), and average variance extracted (AVE), whereas discriminant validity was assessed using construct correlations and the heterotrait–monotrait ratio (HTMT). Measurement invariance was examined across gender because subgroup sample sizes were adequate for multi-group analysis. Educational-level invariance was not tested because the postgraduate subgroup was too small to permit reliable estimation. After acceptable measurement properties were established, structural equation modeling (SEM) was used to test the direct, specific indirect, and sequential indirect associations in the full sample. Indirect effects were evaluated using bias-corrected bootstrapping with 5,000 resamples and were considered statistically significant when the 95% confidence interval did not include zero. Harman's single-factor test was used only as a preliminary check for common method variance. Structural-model results were interpreted in relation to the prespecified hypotheses, standardized path coefficients, explained variance (*R*^2^), and bootstrap-derived indirect effects.

## Results

### Psychometric evaluation of the adapted measures

Parallel analysis and scree-plot inspection in the exploratory subsample (*n* = 283) supported the theoretically specified factor structures of the adapted measures: four first-order factors for psychological capital (musical self-efficacy, musical hope, musical resilience, and musical optimism); a single factor for meaning in music, represented by four indicators (purpose, personal significance, identity relevance, and alignment with valued life goals); a single factor for flow experience, represented by four indicators (absorption, focused attention, fluent action, and perceived challenge–skill balance); and three first-order factors for music-related burnout (music-related exhaustion, music-related cynicism, and diminished musical efficacy). Item loadings ranged from.52 to 0.86. Two items showed modest cross-loadings above the prespecified criterion of 0.30. Specifically, one musical hope item loaded 0.64 on its intended factor and 0.33 on musical optimism, whereas one music-related cynicism item loaded 0.62 on its intended factor and 0.35 on diminished musical efficacy. Both items were retained because their primary loadings were substantial (>0.50) and clearly exceeded their secondary loadings. In addition, the cross-loadings were theoretically interpretable given the conceptual overlap between hope and optimism and between cynicism and diminished efficacy, respectively. Their removal would have reduced the content coverage of the respective constructs without a clear theoretical gain. In the confirmatory subsample (*n* = 282), the retained measurement model demonstrated good fit, χ^2^(344) = 621.47, *p* < 0.001, CFI = 0.951, TLI = 0.945, RMSEA = 0.054, 90% CI [.048,0.061], and SRMR =0.049. A summary of standardized factor loadings, reliability coefficients, composite reliability, and average variance extracted is presented in Supplementary Table 2.

Measurement invariance was examined across gender. The model supported invariance through the scalar level, with changes in fit indices remaining within the recommended thresholds (ΔCFI ≤ 0.010; ΔRMSEA ≤ 0.015). Detailed model-fit comparisons are reported in Supplementary Table 3. Measurement invariance across educational level was not examined because the postgraduate subgroup was too small (n = 12) to support reliable multi-group analysis.

### Common method bias

Harman's single-factor test yielded a Kaiser–Meyer–Olkin value of 0.895, and Bartlett's test of sphericity was significant, χ^2^(741) = 13,268.35, *p* < 0.001. An unrotated principal component analysis of the 39 items identified nine factors with eigenvalues greater than 1, together explaining 71.84% of the total variance. The first factor accounted for 19.01% of the variance. Thus, the test did not indicate a dominant single factor. However, Harman's test cannot rule out common method variance, and the findings may still be affected by shared-source and same-occasion measurement.

### Descriptive statistics and correlations

Table 2 presents the means, standard deviations, distributional properties, internal consistency coefficients, and bivariate correlations for the study variables. All skewness and kurtosis values were below 2.00 in absolute value, indicating no substantial departures from univariate normality. All measures demonstrated satisfactory to excellent internal consistency, with Cronbach's α coefficients ranging from 0.888 to 0.925. Psychological capital was positively correlated with meaning in music, *r* = 0.180, *p* < 0.001, and flow experience, *r* = 0.192, *p* < 0.001, and negatively correlated with music-related burnout, *r* = −0.137, *p* = 0.001. Meaning in music was positively correlated with flow experience, *r* = 0.591, *p* < 0.001, and negatively correlated with music-related burnout, *r* = −0.157, *p* < 0.001. Flow experience was also negatively correlated with music-related burnout, *r* = −0.120, *p* = 0.004.

**Table 2:** Descriptive statistics, internal consistency, and bivariate correlations.

Variable	M	SD	α	1	2	3	4
1.Psychological capital	3.28	0.66	0.913				
2. Meaning in music	2.52	1.08	0.905	0.180^***^			
3. Flow experience	3.62	1.01	0.888	0.192^***^	0.591^***^		
4. Music-related burnout	3.58	0.69	0.925	−0.137^**^	−0.157^***^	−0.120^**^	

*N* = *565*. α = *Cronbach's alpha*. ^**^ *p*<*0.01*. ^***^ *p*<*0.001*.

### Structural equation modeling

The hypothesized structural model demonstrated good fit to the data, χ^2^/df = 1.89, CFI = 0.953, TLI = 0.949, RMSEA = 0.040, 90% CI [0.036, 0.043], and SRMR = 0.044. As shown in Table 3 and Figure 2, psychological capital was positively associated with meaning in music (β = 0.128, *p* = 0.013), supporting H1, and with flow experience *(*β =0.135, *p* = 0.002), supporting H2. Meaning in music was positively associated with flow experience (β = 0.648, *p* < 0.001). The direct association between psychological capital and music-related burnout was negative but nonsignificant (β = −0.028, *p* = 0.618); therefore, H3 was not supported. Meaning in music was negatively associated with music-related burnout (β = −0.145, *p* =0.044), and flow experience was also negatively associated with music-related burnout (β = −0.117, *p* = 0.004).

**Table 3:** Structural path estimates for the hypothesized model.

Structural path	*B*	*SE*	β	*p*	95% CI for *B*	Result
Psychological capital → Meaning in music	0.323	0.130	0.128	0.013	[0.068, 0.577]	Supported
Psychological capital → Flow experience	0.300	0.095	0.135	0.002	[0.113, 0.486]	Supported
Meaning in music → Flow experience	0.572	0.040	0.648	< 0.001	[0.493, 0.651]	Supported
Psychological capital → Music-related burnout	−0.041	0.081	−0.028	0.618	[−0.200, 0.119]	Not supported
Meaning in music → Music-related burnout	−0.083	0.041	−0.145	0.044	[−0.164, −0.002]	Supported
Flow experience → Music-related burnout	−0.076	0.026	−0.117	0.004	[−0.128, −0.024]	supported

**Figure 2:**
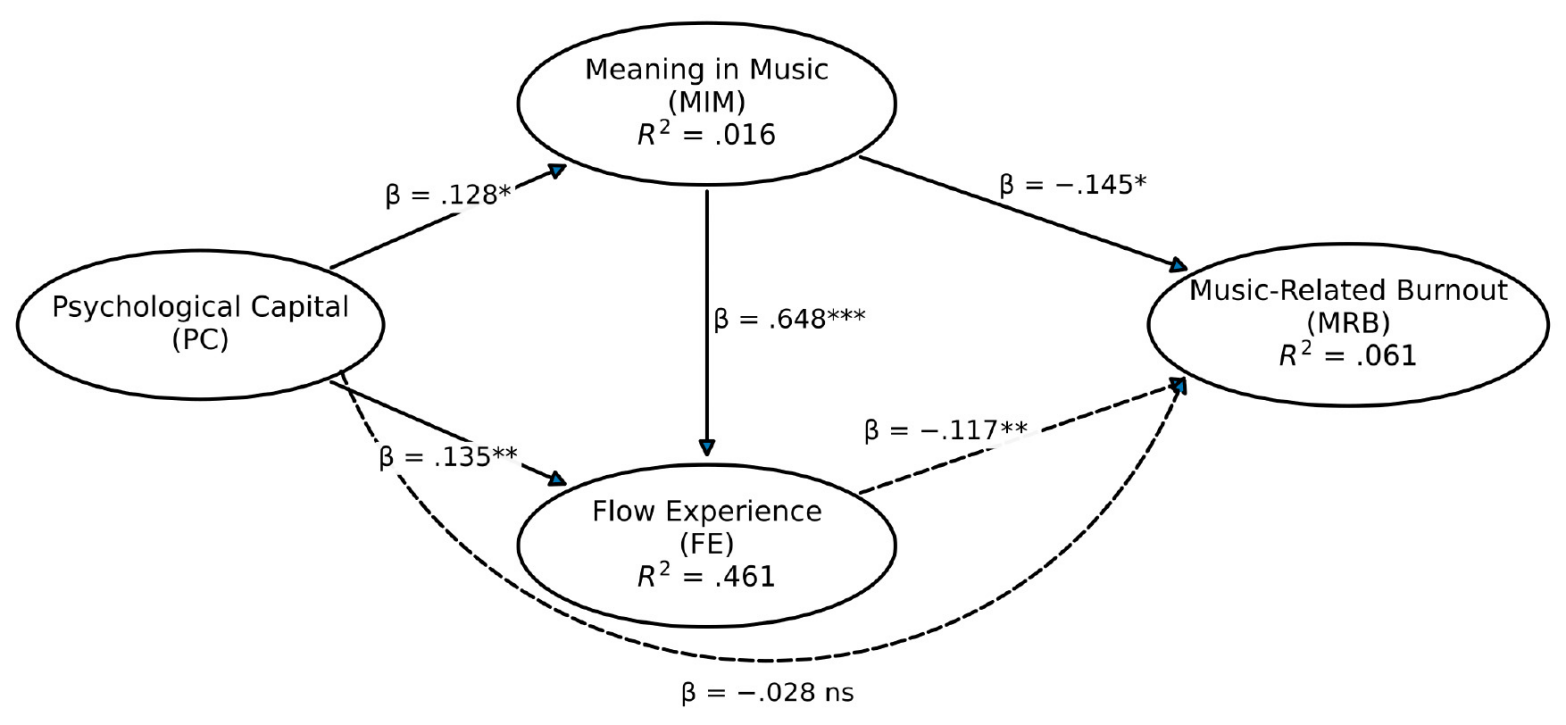
Standardized structural model of the relationships among psychological capital, meaning in music, flow experience, and music-related burnout. Note. Values are standardized path coefficients. Solid lines indicate significant paths; *p <0.05, **p <0.01, ***p <0.001; ns, nonsignificant.

### Assessment of indirect effects

As shown in Table 4, the specific indirect association between psychological capital and music-related burnout through meaning in music was significant (B = −0.027, bootstrap 95% CI [−0.057, −0.008]), supporting H4a. The specific indirect association through flow experience was also significant (B = −0.023, bootstrap 95% CI [−0.047, −0.004]), supporting H4b. The sequential indirect association through meaning in music and flow experience was significant as well (B = −0.014, bootstrap 95% CI [−0.037, −0.003]), supporting H4c. The total indirect effect was statistically significant (B = −0.064, bootstrap 95% CI [−0.086, −0.042]), whereas the direct effect of psychological capital on music-related burnout was not significant (B = −0.041, 95% CI [−0.200, 0.119]). The total effect was also non-significant (B = −0.105, SE = 0.123, 95% CI [−0.346, 0.136]).

**Table 4:** Bootstrap estimates of the indirect effects.

Indirect pathway	*B*	Boot SE	Effect	Boot 95% CI	Result
Psychological capital → Meaning in music → Music-related burnout	−0.027	0.012	−0.027	[−0.057, −0.008]	Supported
Psychological capital → Flow experience → Music-related burnout	−0.023	0.011	−0.023	[−0.047, −0.004]	Supported
Psychological capital → Meaning in music → Flow experience → Music-related burnout	−0.014	0.008	−0.014	[−0.037, −0.003]	Supported
Total indirect effect	−0.064	0.015	−0.064	[−0.086, −0.042]	Significant
Direct effect	−0.041	0.081	−0.041	[−0.200, 0.119]	Not significant
Total effect	−0.105	0.123	−0.105	[−0.346, 0.136]	Not significant

## Discussion

This study examined the association between psychological capital and music-related burnout and evaluated the specific and sequential indirect roles of meaning in music and flow experience among university music students. Consistent with the hypothesized model, psychological capital was positively associated with meaning in music and flow experience, and meaning in music was positively associated with flow experience. Meaning in music and flow experience were each negatively associated with music-related burnout, and all three specific indirect associations were statistically significant. However, both the direct and total effects of psychological capital on music-related burnout were non-significant. Accordingly, the findings should not be interpreted as evidence of an overall or causal protective effect of psychological capital on burnout. Rather, they are consistent with the possibility that psychological capital is linked to music-related burnout through more proximal, domain-specific psychological processes. This interpretation extends previous work by illustrating how broader psychological resources may be associated with students' appraisals and experiences of music learning and performance.

The nonsignificant direct association between psychological capital and music-related burnout suggests that general positive psychological resources may not translate automatically into lower burnout in the music domain. Instead, their relevance may depend on more proximal ways in which students appraise and experience music learning, practice, rehearsal, and performance. In the present model, these domain-specific processes involve meaning-related appraisals and flow-related experiences. Meaning in music concerns whether musical engagement is experienced as purposeful, personally significant, relevant to one's identity, and aligned with valued life or career goals. Flow experience concerns more immediate experiential processes, including focused attention, absorption, fluent action, and a perceived balance between challenge and skill. Psychological capital may be relevant to these processes because confidence, goal-directed motivation, resilience following setbacks, and positive expectations may shape how students interpret demanding musical experiences and how effectively they remain engaged with them. Thus, the findings are consistent with the possibility that psychological capital is associated with burnout when these broader psychological resources are translated into adaptive appraisals and experiences within the specific context of musical activity. Given the cross-sectional design, however, these processes should be interpreted as theoretically plausible explanations of the observed associations rather than as established causal mechanisms.

The indirect association through meaning in music may reflect the motivational relevance of perceiving musical effort as personally worthwhile. University music education involves substantial repetition, delayed improvement, critical evaluation, performance errors, and uncertainty about future outcomes. When students perceive these demands as connected to valued artistic, personal, or professional goals, effort and setbacks may be interpreted as meaningful investments in longer-term development rather than solely as psychological costs. Such appraisals may help sustain motivation and emotional engagement when immediate rewards are limited and may reduce the tendency to disengage from music following difficulties. Meaning should not, however, be regarded as uniformly protective or temporally fixed. Students' perceived meaning in music may change as a function of progress, feedback, performance experiences, autonomy, competing demands, and changing expectations about a musical career. Accordingly, the measure used in the present study is better understood as an assessment of perceived meaning at the time of measurement rather than as an enduring trait. This distinction is important because fluctuations in meaning may themselves be relevant to burnout: periods in which musical activity feels less connected to personally valued goals may coincide with greater exhaustion or disengagement. Conversely, burnout may also reduce students' capacity to experience musical activities as meaningful. The present cross-sectional data cannot distinguish among these possibilities.

Flow may represent a complementary and more immediate experiential process. Whereas, meaning concerns why continued musical engagement is considered worthwhile, flow concerns how that engagement is experienced while it is occurring. Greater psychological capital may be associated with stronger perceived competence, more adaptive responses to errors, sustained goal-directed attention, and a greater willingness to approach demanding tasks, all of which may facilitate focused and absorbed engagement when musical challenges are perceived as manageable. Such experiences may make practice and performance more intrinsically rewarding and may temporarily counter feelings of depletion and disengagement. The significant sequential indirect association is therefore consistent with a purpose-to-absorption interpretation: perceiving musical activity as personally meaningful may provide a motivational basis for sustained investment, while flow may represent an experiential state through which that investment becomes absorbing and rewarding. Importantly, this sequence remains a theoretical interpretation rather than evidence of temporal ordering.

A key theoretical contribution of the present study is the integration of meaning in music and flow experience within a sequential indirect pathway. Although previous studies have examined associations involving psychological capital, meaning, flow, and burnout separately, their joint relationships have received limited attention in the context of music education. The present findings are consistent with a process-oriented account in which psychological capital is associated with stronger meaning in music, which is in turn associated with greater flow experience and lower music-related burnout. This pattern is conceptually coherent because meaning and flow represent distinct but complementary aspects of musical engagement. Meaning in music concerns why students are willing to invest sustained effort in musical development by providing a sense of purpose and personal value, whereas flow concerns how that engagement is experienced during practice and performance. In this sense, perceived meaning may provide a motivational foundation that increases opportunities for flow. The findings therefore extend burnout research, which has largely emphasized performance anxiety, perfectionism, workload, and career uncertainty among music students (Jääskeläinen, 2022), by highlighting positive psychological processes that may be relevant to sustained engagement in music learning.

The findings also suggest several practical implications for higher music education. Rather than focusing solely on workload reduction or general stress-management skills, burnout-prevention initiatives may benefit from combining support for students' psychological capital with attention to domain-specific psychological experiences. For example, instructors can help students set meaningful long-term musical goals, reflect on their artistic values, and recognize progress following setbacks. Instructional conditions that support autonomy, provide constructive feedback, establish clear goals, and maintain an appropriate balance between challenge and skill may also facilitate flow during music practice and performance. Purpose-oriented teaching may further help students connect repetitive technical training with personally valued artistic goals, potentially translating broader psychological resources into more meaningful and fulfilling musical engagement. Because the present study is cross-sectional, these strategies should be viewed as theoretically informed targets for future intervention research rather than as established effective interventions.

Several limitations should be acknowledged. First, the cross-sectional design does not permit conclusions regarding the temporal ordering or causal relationships among psychological capital, meaning in music, flow experience, and music-related burnout. Longitudinal, daily-diary, experience-sampling, and intervention studies are needed to examine whether changes in psychological capital are followed by changes in meaning in music and flow experience and whether these changes are subsequently associated with variation in music-related burnout. Such designs would also help distinguish between-person differences from within-person fluctuations in meaning in music and flow experience. Second, all variables were assessed using self-report measures, which may introduce common method variance. Although Harman's single-factor test did not indicate a dominant single factor, shared-method effects cannot be ruled out. Third, convenience sampling and recruitment exclusively from 10 universities in Shandong Province may limit the generalizability of the findings. The sample may not adequately represent music students from other regions of China or from different cultural and educational contexts. Future studies should therefore recruit more geographically diverse samples and, where feasible, employ probability-based or stratified sampling approaches. In addition, the small and uneven numbers of participants at more advanced educational stages, particularly in the postgraduate subgroup, prevented reliable comparisons across educational levels. Future studies with larger and more balanced educational-stage samples should examine whether developmental factors, such as coping experience, self-regulation, professional identity, and career concerns, are associated with music-related burnout and its proposed psychological correlates. Finally, other potentially relevant contributors to music-related burnout, including perfectionism, practice intensity, teacher support, and performance anxiety, were not included in the present model and should be considered in future research.

## Conclusion

The present findings indicate that specific indirect associations linked psychological capital with music-related burnout through meaning in music and flow experience, despite nonsignificant direct and total effects. This pattern highlights the potential relevance of domain-specific psychological processes for understanding how broader positive psychological resources relate to students' experiences of musical engagement and burnout. By integrating psychological capital with meaning in music and flow experience, the study offers a more differentiated account of burnout among university music students and identifies theoretically informed targets for future longitudinal and intervention research. Given the cross-sectional design, causal and temporal interpretations should remain tentative.

## Data Availability

The original contributions presented in the study are included in the article/supplementary material, further inquiries can be directed to the corresponding author/s.
