# Supplementary material for: Psychological capital and music-related burnout among university music students: the mediating effects of meaning in music and flow experience
Source: Front Psychol. 2026 Oct 14;17:1956838. doi: 10.3389/fpsyg.2026.1956838 (PMC13713336; doi:10.3389/fpsyg.2026.1956838)
Supplement: Supplementary file 1 [file Table_1.docx]

**Supplementary Table**

Supplementary Table S1 Reliability and Convergent Validity of Meaning in Music, Flow Experience, and Music-Related Burnout

| Dimension | Items | Cronbach’s α | McDonald’s ω | CR | AVE |
| --- | --- | --- | --- | --- | --- |
| Musical self-efficacy | 4 | .875 | .879 | .881 | .650 |
| Musical hope | 4 | .862 | .866 | .868 | .622 |
| Musical resilience | 4 | .846 | .851 | .853 | .593 |
| Musical optimism | 4 | .858 | .863 | .865 | .616 |
| Psychological capital(overall) | 16 | .913 | .918 | .921 | .594 |
| Meaning in Music | 4 | .905 | .907 | .907 | .710 |
| Flow experience | 4 | .888 | .889 | .889 | .667 |
| Exhaustion | 5 | .888 | .888 | .888 | .612 |
| Cognitive disengagement | 5 | .900 | .901 | .901 | .645 |
| Emotional impairment | 5 | .884 | .885 | .885 | .606 |
| Music-related burnout (overall) | 15 | .925 | .925 | .925 | .451 |

Supplementary Table S2 Psychometric Properties of the Adapted Measures

| Construct | Retained structure | Loading range | α / ω | CR / AVE | Maximum HTMT |
| --- | --- | --- | --- | --- | --- |
| Psychological capital | 4 first-order factors + 1 second-order factor | .55–.84 | .913 / .918 | .921 / .594 | .82 |
| Meaning in music | 1 factor | .72–.89 | .905 / .907 | .907 / .710 | .79 |
| Flow experience | 1 factor | .69–.86 | .888 / .889 | .889 / .667 | .76 |
| Music-related burnout | 3 first-order factors + 1 second-order factor | .52–.85 | .925 / .925 | .925 / .612 | .84 |
| Psychological capital | 4 first-order factors + 1 second-order factor | .55–.84 | .913 / .918 | .921 / .594 | .82 |
| Meaning in music | 1 factor | .72–.89 | .905 / .907 | .907 / .710 | .79 |
| Flow experience | 1 factor | .69–.86 | .888 / .889 | .889 / .667 | .76 |
| Music-related burnout | 3 first-order factors + 1 second-order factor | .52–.85 | .925 / .925 | .925 / .612 | .84 |
| Psychological capital | 4 first-order factors + 1 second-order factor | .55–.84 | .913 / .918 | .921 / .594 | .82 |
| Meaning in music | 1 factor | .72–.89 | .905 / .907 | .907 / .710 | .79 |
| Flow experience | 1 factor | .69–.86 | .888 / .889 | .889 / .667 | .76 |

Supplementary Table S3 full item-level results.

| Model | χ² | df | CFI | RMSEA | ΔCFI | ΔRMSEA |
| --- | --- | --- | --- | --- | --- | --- |
| Gender: Configural | 1243.61 | 688 | .950 | .053 | — | — |
| Metric invariance | 1268.34 | 716 | .949 | .052 | -.001 | -.001 |
| Scalar invariance | 1301.76 | 744 | .946 | .053 | -.003 | .001 |
| Strict invariance | 1378.92 | 781 | .935 | .057 | -.011 | .004 |
| Educational stage: Configural | 1251.43 | 688 | .949 | .054 | — | — |
| Metric invariance | 1274.85 | 716 | .947 | .054 | -.002 | .000 |
